# Time-Dependent Structural Alteration of Rituximab Analyzed by LC/TOF-MS after a Systemic Administration to Rats

**DOI:** 10.1371/journal.pone.0169588

**Published:** 2017-01-04

**Authors:** Yuki Otani, Atushi Yonezawa, Masahiro Tsuda, Satoshi Imai, Yasuaki Ikemi, Shunsaku Nakagawa, Tomohiro Omura, Takayuki Nakagawa, Ikuko Yano, Kazuo Matsubara

**Affiliations:** 1 Department of Clinical Pharmacology and Therapeutics, Kyoto University Hospital, Kyoto, Japan; 2 Graduate School of Pharmaceutical Sciences, Kyoto University, Kyoto, Japan; H Lee Moffitt Cancer Center and Research Institute, UNITED STATES

## Abstract

Therapeutic monoclonal antibodies (mAbs) have heterogeneities in their structures. Multiple studies have reported that the variety of post-translational modifications could affect the pharmacokinetic profiles or pharmacological potencies of therapeutic mAbs. Taking into the account that the structural modification of mAbs would affect the efficacy, it is worth investigating the structural alteration of therapeutic mAbs in the blood and the relationship between their structures and pharmacological effects. Herein, we have developed the method to isolate rituximab from plasma in which endogenous IgGs interfere the detection of rituximab, and successfully developed the analytical method with a liquid chromatograph time-of-flight mass spectrometer to detect the structure of rituximab in plasma with errors less than 30 parts per millions. Eight types of carbohydrate chains in rituximab were detected by this method. Interestingly, time-dependent changes in carbohydrate chains such as AAF (G2F) and GnGn (G0) were observed in rats, although the amino acids were stable. Additionally, these structural changes were observed via incubation in plasma as in the rat experiment, suggesting that a certain type of enzyme in plasma caused the alterations of the carbohydrate chains. The present analytical methods could clarify the actual pharmacokinetics of therapeutic mAbs, and help to evaluate the interindividual variations in pharmacokinetics and efficacy.

## Introduction

Therapeutic monoclonal antibodies (mAbs) have made a breakthrough in the treatment of cancer, autoimmune diseases, asthma and so on. The advantages of therapeutic mAbs are their high specificities for target molecules and their long half-lives [1]. Recent antibody engineering has enabled therapeutic mAbs to elicit potent pharmacological effects and reduce immunogenicity [1]. However, precision medicine with therapeutic mAbs remains a challenge as yet. The therapeutic effects of mAbs are influenced by multiple factors such as the plasma or tissue concentrations of therapeutic mAbs, the amounts of antigens expressed on cancer cells, and the immune state of patients [2]. In this study, we focused on pharmacokinetics of therapeutic mAbs, because there are many ambiguous factors lacking analytical technologies. In the cases treated with low-molecular weight therapeutic agents, we can obtain clinical data on the blood concentrations of parent compounds and metabolites using a liquid chromatography-mass spectrometer. Currently, an enzyme-linked immunosorbent assay (ELISA) is general method that has been extensively applied for measuring blood concentrations of therapeutic mAbs. Recently, numerous efforts have been made to develop another quantification method of mAbs using LC/MS/MS [3, 4]. On the other hand, a robust method to assess structures of therapeutic mAbs in the body has not been developed to date in spite of their structural heterogeneities [5].

The structural complexity of therapeutic mAbs is mainly caused by their post-translational modifications. Multiple studies have reported that the variety of post-translational modification could affect the pharmacokinetic profiles and/or pharmacological effects of therapeutic mAbs [6, 7]. It is well known that afucosylation of the carbohydrate chain largely increases antibody-dependent cellular cytotoxicity (ADCC) activity owing to its higher affinity to Fcγ receptor [8–10], and this improves clinical outcome [11, 12]. Post-translationl modifications of therapeutic mAbs should also affect their clearance. A high-mannose type glycan in the fragment crystallizable region increases the IgG clearance rate in humans due to cellular uptake via the mannose receptor [13, 14].

Taking into account of the fact that the structural modification of mAbs would affect its efficacy, it is worth investigating the structural alteration of therapeutic mAbs in the blood and the relationship between their structures and pharmacological effects. Indeed, Chu et al. have previously reported that modifications of bevacizumab in rabbit eyes are detected by using a high performance liquid chromatography [15]. Moreover, it has been published that alterations of the glycans in mAbs are detected in vivo experiments [16, 17]. However, these reports have mentioned the alteration of only a part of the structures such as the amino acids or glycans. In order to develop precision medicine with therapeutic mAbs, it is necessary to understand the pharmacokinetics of therapeutic mAbs including structural alterations in the same way as low-molecular weight therapeutic agents.

**Fig 1:**
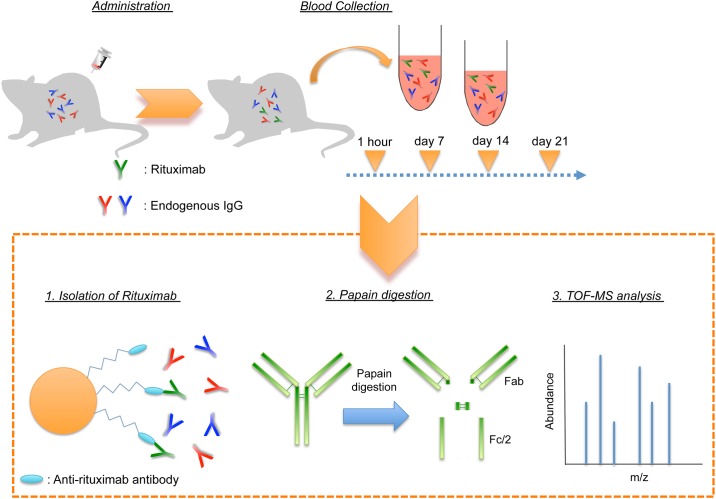
Schematic representation of present method for measuring rituximab in plasma. Isolation of rituximab from plasma was conducted by anti-rituximab antibodies covalently bound to magnetic beads. Isolated rituximab was digested by papain, and then the structures of rituximab were analyzed by LC/TOF-MS.

In the present study, we have developed a method to analyze the structure of rituximab (Rituxan^®^, Chugai Pharmaceutical Co., Ltd., Tokyo, Japan) in vivo, as a model for therapeutic mAbs using a liquid chromatograph time-of-flight mass spectrometer (LC/TOF-MS). Rituximab was isolated from plasma using affinity magnetic beads coated with anti-rituximab mAb, followed by digesting these through the use of the papain to obtain fragment antigen-binding (Fab) and reduced fragment crystallizable (Fc/2). The isolated and digested rituximab was analyzed with LC/TOF-MS in order to observe the structure of therapeutic mAb including carbohydrate chains in the plasma (Fig 1). In addition, we determined complement-dependent cytotoxicity (CDC) and ADCC activities of the rituximab, which had been isolated from plasma.

## Materials and Methods

### Animals

Wistar/ST rats (7-week, male) were purchased from Japan SLC, Inc. (Shizuoka, Japan). All animal experiments were performed in accordance with the Principles of Laboratory Animal Care as adopted and propagated by the U.S. National Institutes of Health and the Guidelines for Animal Experiments of Kyoto University. All protocols were approved by the Animal Research Committee, Graduate School of Medicine, Kyoto University (MedKyo16064).

### Pharmacokinetic experiment of rituximab

Rituximab (Chugai Pharmaceutical Co., Ltd., Lot: E9103AA) was administered to anesthetized rats at 10 mg/kg via the jugular vein. At the designated times, blood samples were collected with heparin-coated syringes via the abdominal aorta. Blood samples were immediately centrifuged at 3,000 rpm for 15 min to obtain plasma.

### Isolation of rituximab from plasma

Isolation of rituximab was performed with M-280 tosylactivated magnetic beads (Life Technologies, California, USA) according to the protocol provided by the supplier. In brief, 200 μg of anti-rituximab antibody (MB2A4) (Gene Tex, California, USA) were covalently attached to 10 mg of magnetic beads. Then, the free tosylate groups of the beads were blocked by incubation with PBST containing 1% BSA for 1 h. Rat plasma samples were concentrated using an Amicon^®^ Ultra 100K filter (Merck Millipore, Massachusetts, USA). The concentrated plasma was incubated with the beads for 3 h at room temperature. After washing, rituximab was eluted with 0.1 M glycine-HCl (pH 2.7).

For the CDC and ADCC assays, plasma samples were purified to isolate rituximab by an Ex-Pure Spin ProA (Kyoto Monotech, Kyoto, Japan) centrifuge following the protocol provided by the supplier.

### Analysis of rituximab by LC/TOF-MS

Papain (Roche Applied Science, Penzberg, Germany) was diluted to 1 mg/mL solution with phosphate buffer (pH 7.0) containing 10 mM cysteine. Immediately, the solution was incubated for 10 min at 37°C. Following activation of the papain, cysteine was removed from the solution by ultrafiltration with an Amicon^®^ Ultra 10K filter (Merck Millipore, Massachusetts, USA). Subsequently, activated papain was added to the rituximab solution at a final concentration of 15% (w/w), and incubated for 8 h at 37°C. At the end of the reaction, 3% (v/v) of 0.5 M iodoacetoamide was added to the mixture.

The digested rituximab was analyzed by an Agilent 1200 series LC system coupled with an Agilent 6530 Accurate-Mass Q-TOF LC/MS system (Agilent Technologies, California, USA). The LC conditions used were as follows: column, Poroshell 300SB C8 column (2.1×75 mm, 5 μm); mobile phase, 0.1% formic acid and acetonitrile (gradient from 10% to 61% of acetonitrile in 15 min); flow rate, 0.2 ml/min; injection volume, 8 μl; and column temperature, 70°C. Instrument parameters were set as follows: Positive ion mode (m/z range from 300 to 3,200); drying gas (N_2_), 12 L/min, 350°C; sheath gas, 12 L/min, 400°C; nebulizer gas, 60 psig; capillary, 3,500 V; skimmer, 65 V; octopole RF, 750 V; fragmentor, 350 V. As calibration standards, HP-921 and HP-1221 (Agilent Technologies, California, USA) were used during measurements.

Data were analyzed using MassHunter Workstation Software Qualitative Analysis B. 06. 00 (Agilent Technologies, California, USA). Then the mass spectra were deconvoluted by the maximum entropy method to calculate molecular weights of these fragments. Relative abundance of each glycoform was represented by the ratio of each peak height in deconvoluted mass spectrum.

### In-vitro incubation of rituximab

Heat-inactivated plasma was obtained by the incubation at 56°C for 30 min. Rituximab was incubated in rat plasma, heat-inactivated plasma or phosphate buffer at 37°C for up to 21 days. After the incubation, rituximab was isolated from each sample and analyzed by LC/TOF-MS according to the methods described above.

### Determination of plasma concentration by ELISA

Rituximab concentration was determined by ELISA according to the previous report [18]. In brief, 1 μg/mL of rat anti-rituximab antibody (MB2A4) in coating buffer (15 mM sodium carbonate and 28.5 mM sodium bicarbonate at pH 9.6) was added to each well of Nunc-Immuno^™^ MicroWell^™^ 96 well solid plates (Sigma-Aldrich^®^, Missouri, USA), then reacted overnight at 4°C. The coated plate was blocked, and samples were added to the each well followed by incubation for 1 h. Chicken anti-human IgG (Fab) antibody labeled with horseradish peroxidase (HRP) (Thermo Fisher Scientific, Massachusetts, USA) was diluted at 1/50,000, and was then added to the each well to incubate for 90 min. Finally, HRP substrate (KPL, Inc., Maryland, USA) was added followed by measuring absorbance at 450 nm.

### CDC assay of isolated rituximab

Isolated rituximab samples were diluted at designated concentrations with phosphate saline buffer. Raji cells (ATCC, CCL-86) were prepared at a density of 1.0 x 10^6^ cell/mL and plated 50 μL to each well in the 96-well plate. Diluted 40-μL rituximab samples were then added to the wells followed by incubation for 10 min at room temperature. After the incubation, 40 μL of 20-fold diluted human complement (Cedarlane, Ontario, Canada) was added to the wells. Then the plates were incubated for 2 h at 37°C. The number of living cells was determined using cell count reagent SF (Nacalai Tesque, Kyoto, Japan) according to the protocol provided by the supplier. The absorbance at 460 nm was measured by VERSAmax microplate reader. (Molecular Devices, California, USA)

### ADCC assay of isolated rituximab

ADCC activity of isolated rituximab was determined using ADCC Reporter Bioassay Kit (Promega, Wisconsin, USA) following the protocol provided by the supplier. Rituximab and trastuzumab in the formulations were used as a positive control and negative control, respectively. The detection of luminescence was performed by Mithras LB940 (Berthold Technologies, Germany).

### Statistical analysis

Data were expressed as the means ± standard deviation of more than three experiments. Statistical analysis was performed using analysis of variance and the Dunnett’s test for multiple comparisons with control group or Student’s t-test for two-group comparisons. p < 0.05 was considered statistically significant.

## Results

### Analysis of digested rituximab by LC/TOF-MS

**Fig 2:**
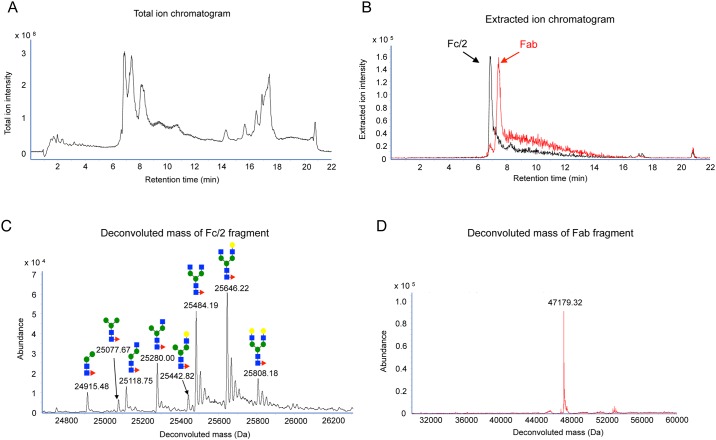
LC/TOF-MS analysis of digested rituximab in the formulation. (A) Total ion chromatogram of the rituximab formulation that was digested by papain. The peaks at 6.8 and 7.3 min were Fc/2 and Fab fragments, respectively. (B) Overlaid extracted ion chromatograms at 1,509 m/z and 1,475 m/z, which corresponded to the Fc/2 and Fab fragments ions, respectively. (C) Typical deconvoluted chromatogram of Fc/2 fragment of the rituximab formulation. The number and pattern diagram above each peak indicate the observed molecular weight and predicted structure of attached carbohydrate chains, respectively. (D) Typical deconvoluted chromatogram of the Fab fragment of the rituximab formulation. The observed molecular weight of the Fab fragment is indicated above the peak.

**Fig 3:**
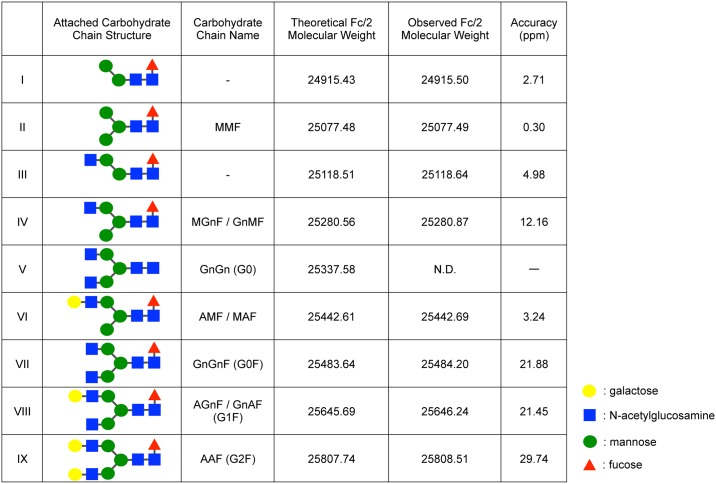
Detected glycoforms in rituximab and the predictive carbohydrate chains. The carbohydrate chains were named according to the proglycan system (www.proglycan.com). Note that Fc/2 molecular weights were the mean values of three independent experiments. The accuracies of these experiments were expressed in terms of part per million (ppm).

To start this study with LC/TOF-MS, we optimized analytical conditions of the instrument using commercial formulations of rituximab. Two peaks at 6.8 and 7.3 min on the total ion chromatogram were identified as Fc/2 and Fab fragments of rituximab, respectively (Fig 2A). Full widths at half maximum of these peaks were clearly separated in each extracted ion chromatogram (Fig 2B). Since two mass spectra obtained from these peaks did not interfered with each other, deconvolution analysis to obtain their molecular weights was separately conducted with spectra from these peaks. In the deconvoluted spectrum of the Fc/2 fragment, eight peaks were identified as each of the glycoforms on the basis of their molecular weights and the previous reports [19, 20] (Fig 2C). The predictive structures and nomenclatures of carbohydrate chains attached to each glycoforms (I-IX) are summarized in Fig 3. The observed molecular weights of glycoforms were obtained at accuracies of within 30 ppm. The reproducibility was also confirmed by assessing inter-day and intra-day variations (S4 Fig). Furthermore, only one peak corresponding to Fc/2 fragment was detected in deglycosylated rituximab by Endo S, which is an endoglycosidase with a high specificity for removing N-linked glycans from the chitobiose core of the heavy chain of native IgG (S2 Fig), and its molecular weight was 24,388.10 Da. Because the theoretical molecular weight of a deglycosylated Fc/2 fragment is 24,500.35 Da, commercial formulations of rituximab used in this study had undergone a deletion of C-terminal lysine residue (-128 Da) and an oxidation at one of methionine residues (+16 Da) in the manufacturing process [21–23]. On the other hand, the peak at 7.3 min was identified as Fab fragments, and its molecular weight was calculated 47,179.32 Da by deconvolution analysis (Fig 2D). Any modifications were not observed in Fab fragment.

We isolated rituximab from rat plasma using anti-rituximab antibody, and compared mass spectra between rituximab in the formulation and isolated molecule. As shown in S3A and S3B Fig, the Fc/2 mass spectrum of the molecule isolated from plasma was the same as that of rituximab in the formulation (Fig 2C). Eight types of glycoforms were also observed in the deconvoluted mass spectra. Moreover, peaks of the Fab fragments isolated from the commercial formulation and spiked plasma were observed at 47,179.06 and 47,179.19 Da, respectively (S3C and S3D Fig). These results indicated that the process of rituximab isolation had no impact on the analysis of rituximab by LC/TOF-MS.

### Assessment of structural alteration of rituximab in rats

**Fig 4:**
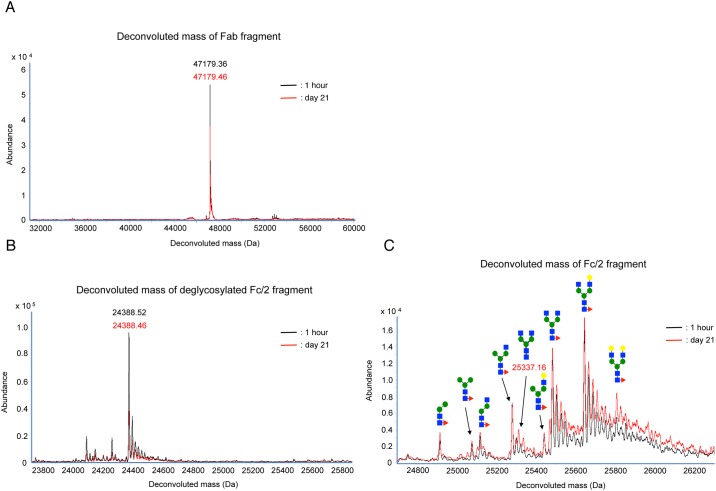
LC/TOF-MS analysis of isolated rituximab from rat plasma. The mass spectra were overlaid to compare 1-h and day-21 samples. Black and red lines indicated the mass spectra of rituximab isolated from the samples of 1 h and day 21, respectively. The number and pattern diagram above each peak indicate observed molecular weight and predicted structure of attached carbohydrate chains, respectively. Typical deconvoluted mass spectra of Fab (A), deglycosylated Fc/2 (B) and Fc/2 (C) fragments of rituximab.

We investigated the time-dependent structural alteration of rituximab in vivo. Rituximab in the commercial formulation (10 mg/kg) was administered intravenously to Wistar/ST rats. On 21 days of post-administration, plasma concentration of rituximab remained over 10 μg/ml, which was enough level to analyze the molecular structure (S1 Fig). Thereafter, rituximab in the plasma was isolated, and its structures were analyzed. The deconvoluted masses of Fab fragments were not significantly different between the isolated samples collected at 1 h and on 21 days after the administration (Fig 4A). We also determined the structural alteration in Fc/2 fragments. Molecular weights of deglycosylated Fc/2 fragments from the isolated rituximab remained the same in the plasma collected at 1 h and on 21 days after administration (Fig 4B). We compared Fc/2 fragment including its carbohydrate chains in the samples at 1 h and on 21 days of post-administration. In addition to 8 peaks observed in the sample at 1 h, a newly appeared peak at molecular weight of 25,337.16 Da was detected at day 21 (Fig 4C).

**Fig 5:**
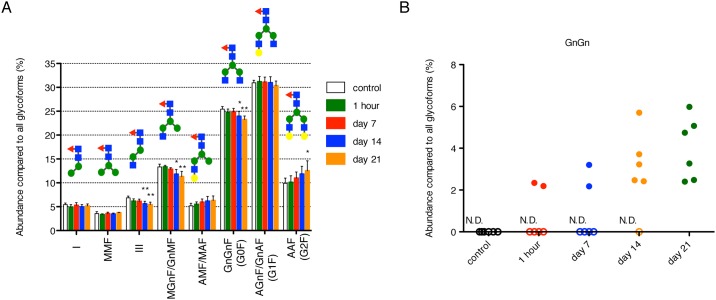
Time-dependent changes in composition ratios of rituximab glycoforms in rat. These values were calculated from peak heights of each glycoform from deconvoluted mass spectrum. Control represents isolated rituximab from plasma spiked with the formulation. (A) Each datum represents the mean of six independent replicates with standard deviation. The composition ratios of glycoforms of III, MGnF/GnMF (IV) and GnGnF (G0F, VII) (see Fig 3) are significantly different between the samples on 14 or 21 days of post-administration and controls (*: p<0.05, **: p<0.01). Additionally, the composition ratio of IX glycoform is significantly different (*p<0.05) between the samples on 21 days of post-administration and controls. (B) The composition ratios of GnGn (G0) glycoform at each time point. Each point represents the value obtained from individual plasma sample. Open symbol indicates that GnGn was not detected in these samples.

Based on the deconvoluted peak heights of Fc/2 fragments, we quantified the composition ratios of 8 types of glycoforms, which were detected in all plasma samples at any time point (Fig 5). AGnF/GnAF (VIII in Fig 3), generally called as G1F, accounted for approximately 30% in all carbohydrate chains. This composition ratio did not show any significant change until 21 days of post-administration. However, the composition ratio of GnGnF (G0F, VII in Fig 3) significantly reduced on 14 and 21 days of post-administration compared with that in the rituximab formulation in a time-dependent manner. The same profile was observed in the glycoform of MGnF/GnMF (IV in Fig 3) and III (in Fig 3). Conversely, the ratio of AAF (G2F, IX in Fig 3) significantly increased at day 21 compared with control (Fig 5A). Several studies have reported that mAbs, which are converted all glycoforms to GnGn (G0, V in Fig 3), display a 10- to 100-fold increase in ADCC activity in vitro [8, 24]. Thus, it was highly significant to investigate alteration in the composition ratio of GnGn glycoform. Although GnGn glycoform was not detected in 4 out of 6 rats at 1 h of post-administration, this was detected in all rats on 21 days after administration; the mean ratio was approximately 4% (Fig 5B).

### CDC and ADCC activities of isolated rituximab

**Fig 6:**
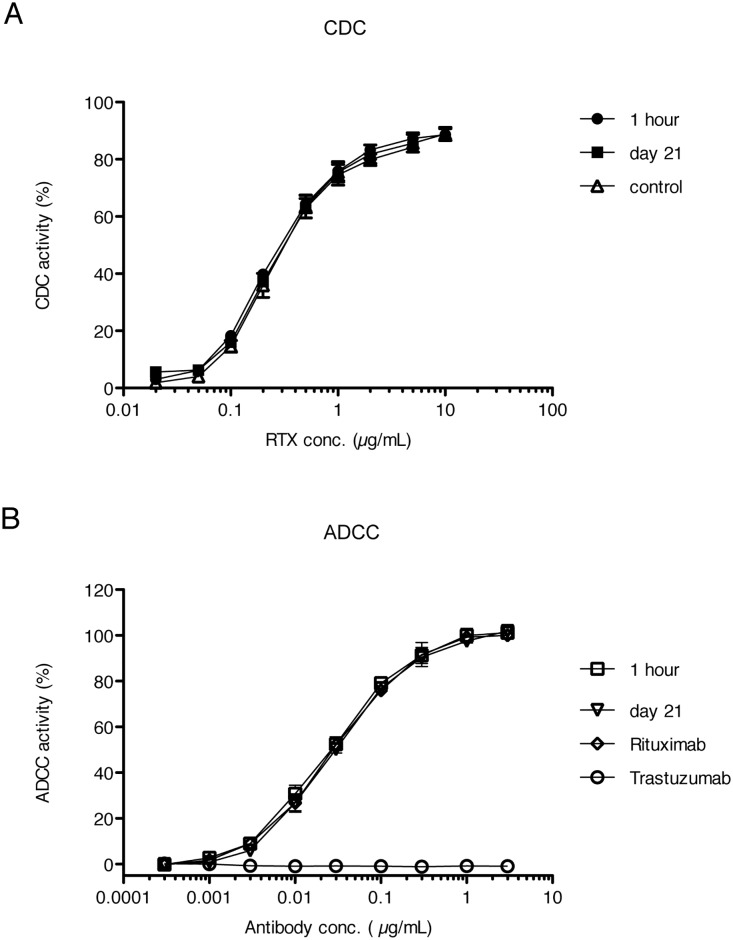
CDC and ADCC activities of rituximab from rat plasma. (A) Complement-dependent cytotoxicity (CDC) activities were determined with rituximab isolated from plasma of 1 h and 21 days of post-administration. Control represents the activity of rituximab in the formulation. (B) Antibody-dependent cellular cytotoxicity (ADCC) activities were determined with rituximab isolated from plasma of 1 h and 21 days of post-administration. Rituximab and Trastuzumab represent the activities of the rituximab and trastuzumab formulations, respectively. Each datum represents the mean of four independent replicates with standard deviation.

We conducted CDC and ADCC assays using rituximab isolated from rat plasma at 1 h and on 21 days after a systemic administration. Also, we determined both activities in the formulations as control values. CDC activities of rituximab did not show any significant differences among plasma samples collected at different times (1 h and 21 days after administration) and the formulation (Fig 6A). Also, ADCC activities of rituximab isolated from rat plasma on day-21 were similar to those from 1 h samples and the rituximab formulation (Fig 6B). These results indicated that glycoform alteration of rituximab in rat plasma did not affect CDC and ADCC activities.

### Assessment of structural alteration of rituximab in plasma

**Fig 7:**
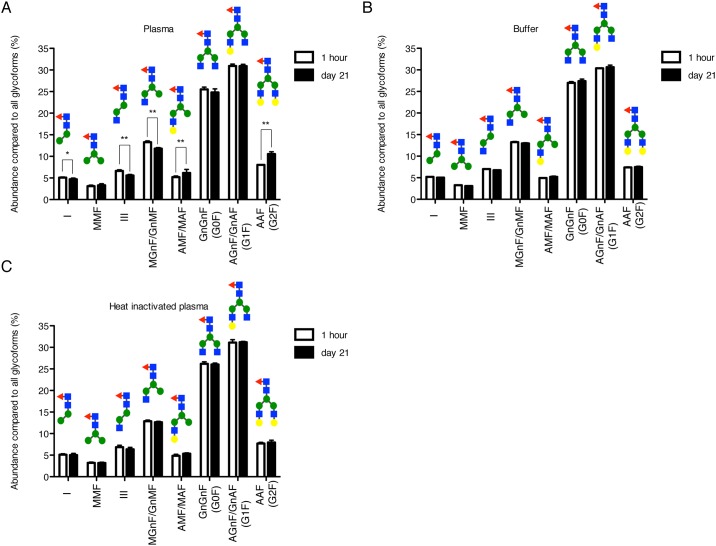
Composition ratios of glycoforms at 1 h and on 21 days after *in vitro* incubation. Rituximab was incubated at 37°C with (A) rat plasma, (B) phosphate buffer and (C) heat-inactivated rat plasma. Each datum represents the mean of five or six independent replicates with standard deviation. In rat plasma, the composition ratios of glycoforms of I, III, IV, VI and IX showed significant differences between 1 h and day 21 (*: p<0.05, **: p<0.01). In the samples of the buffer and heat-inactivated plasma, there is no significant difference between 1-h and day-21 samples.

Following the in vivo experiment, we assessed the alteration of rituximab in plasma. After rituximab was incubated in rat plasma for up to 21 days at 37°C, the composition ratio of carbohydrate chains was slightly changed as shown in the result of the in vivo experiment. The composition ratios of AAF (G2F, IX) and AMF/MAF (VI) significantly increased on 21 days compared with those at 1 h after incubation. Contrary to these phenomena, the composition ratios of I, III and MGnF/GnMF (IV) significantly reduced at 21 days compared with those at 1 h after incubation (Fig 7A). However, no alterations of these carbohydrate chains were observed, when incubated in phosphate buffer (Fig 7B) and in heat-inactivated plasma (Fig 7C). The amino acids, which compose Fab and Fc/2 fragments, were stable in plasma.

## Discussion

Therapeutic mAbs have heterogeneities in their structure [5], while structural alteration of therapeutic mAbs in the body is absolutely unclear. In this study, we have developed a method to isolate rituximab from plasma, in which endogenous IgGs interfere in the analysis of rituximab, and successfully established an analytical method for determining the rituximab structure using LC/TOF-MS with errors less than 30 parts per millions. By the use of this new technique, it was revealed that amino acids, which compose mAbs, were stable, however, certain carbohydrate chains were gradually changed in the body. The plasma concentration of rituximab in clinical trials is measured by ELISA using anti-rituximab antibody (MB2A4) [18, 25], which was used to isolate rituximab from plasma in this study. The use of the present analytical method in combination with ELISA makes it possible to clarify and reveal pharmacokinetic information of therapeutic mAbs in the body.

In many previous studies, glycosylation forms of mAb formulations have been analyzed for quality management. In these studies, the conventional method to analyze the carbohydrate chains of mAbs has been employed; N-glycans or glycopeptides have been released from the mAbs, followed by liquid chromatography-mass spectrometer analysis [26–28]. The present study enabled to assess not only the carbohydrate chains but also other parts of the structure, such as the Fc region excluding carbohydrate chains and the Fab fragment, by the papain digestion and the use of LC/TOF-MS. We used papain to obtain fragments from rituximab in this study, because Adamczyk M et al. reported that papain digestion under cysteine-free condition could homogeneously obtain univalent fragments, Fab and Fc/2 [29]. IdeS, which cleaves heavy chains below the hinge region, also produce also Fc/2 fragment, but cannot digest bivalent F(ab’)_2_ into Fab fragment [30]. Therefore, we chose papain instead of IdeS in this study. Using this method, we detected that rituximab in the commercial formulation received post-translational modifications, that is, the removal of C-terminal lysine residue and oxidation of the methionine residue. These modifications of rituximab in the formulation have been reported previously [21–23]. We detected 8 types of carbohydrate chains in Fc/2 fragment in this study. This fact was also in line with previous reports [19, 31]. These results indicated that the combination of papain digestion and LC/TOF-MS was also a feasible fashion to analyze the structure of therapeutic mAbs for the quality management of formulations.

To our knowledge, it was a new finding that the time-dependent changes occurred in carbohydrate chains such as AAF (G2F) and GnGn (G0) in the body after systemic administration. The same structural alteration was also observed, when rituximab was incubated in plasma, but not in buffer. The composition ratios of some types of carbohydrate chains in rituximab increased, while other types decreased. It was suggested that the alteration of composition ratios was probably caused by conversion from one type of carbohydrate chain to another in the plasma. Previous studies demonstrated that activities of several glycohydrolases of lysosomal origin, such as β-galacotosidase, β-glucosidase and α-fucosidase, maintained even in plasma [32, 33]. In addition, Gmeiner B et al. reported that activities of glycosyltransferases such as galactosyltransferase were detected in several cell lines and their culture media [34]. Taken together with previous reports, the results suggest that the presence of glycohydrolases and/or glycosyltransferases in plasma could be responsible for the alteration of glycoforms in mAbs in plasma.

Rituximab is administered every 21 days in the R-CHOP regimen, which is one of representative regimens for the treatment of non Hodgkin’s lymphoma patients. Although rituximab slightly underwent structural transitions in the rat body, no significant differences in both CDC and ADCC activities were observed up to 21 days after systemic administration. However, there are some plausible reports that serum level of galactosyltransferase associated with tumor increases in patients with bladder [34] and ovarian cancer [35]. Besides, the expression level of fucosyltransferase IV, which is involved in the altered glycosylation of cancer, has significantly increased in breast cancer tissues and serum [36]. These previously reported findings together with our results might lead a hypothesis that structural changes in therapeutic mAbs could have occurred to cause interindividual variations in the efficacy and adverse effects in human. We are currently conducting a clinical study, which has been registered in UMIN-CTR (UMIN000016713), to elucidate the interindividual variations of the structural alterations in rituximab in patients.

In conclusion, we have successfully developed an analytical new method with LC/TOF-MS to detect the structure of therapeutic mAbs in plasma, and determined structural changes in the carbohydrate chains of rituximab in vivo. This carbohydrate chain alteration in the rituximab structure may be due to certain enzymes in plasma. The present analytical methods could clarify the actual pharmacokinetics of therapeutic mAbs, and help to account for the interindividual variations in pharmacokinetics and efficacy.

## Supporting Information

S1 Fig Rat plasma concentration profile of rituximab. Rituximab (10 mg/kg) was administered to anesthetized rats at 10 mg/kg via the jugular vein. The concentration was determined by ELISA coated with anti-rituximab antibody (MB2A4). Each datum point represented the mean of six independent replicates with standard deviation.
(TIF)

S2 Fig Typical deconvoluted chromatogram of Fc/2 fragment of deglycosylated rituximab. Rituximab in the formulation was deglycosylated for 1 h by EndoS. Papain digestion was conducted prior to LC/TOF-MS analysis. The most abundant ion in this spectrum was a deglycosylated Fc/2 fragment of rituximab.
(TIF)

S3 Fig LC/TOF-MS analysis of digested rituximab. (A, B) Typical deconvoluted mass spectra of Fc/2 fragments of rituximab isolated from the commercial formulation and spiked plasma. The number and pattern diagram above each peak indicate observed molecular weights and predicted structures of attached carbohydrate chains, respectively. (C, D) Typical deconvoluted mass spectra of deglycosylated Fab fragments of rituximab isolated from the commercial formulation and spiked plasma. The most abundant ion in each spectrum was the Fab fragment of rituximab.
(TIF)

S4 Fig Intra-day and Inter-day variation of LC/TOF-MS analysis of Fc/2 fragments. Observed Fc/2 molecular weights were the mean values of three independent experiments and the standard deviations of the experiments are given. Detected glycoforms in the rituximab formulation and the predictive attached carbohydrate chains were described in the same way as in Fig 3.
(TIF)

S1 Table Individual values of relative peak heights of each glycoform in Fig 5A. (XLSX)

S2 Table Activity values of CDC and ADCC for each experiment. (XLSX)

S3 Table Individual values of relative peak heights of each glycoform in Fig 7. (XLSX)
