# Supplementary figures and images for: Time-Dependent Structural Alteration of Rituximab Analyzed by LC/TOF-MS after a Systemic Administration to Rats
Source: PLoS One. 2017 Jan 4;12(1):e0169588. doi: 10.1371/journal.pone.0169588 (PMC5215255; doi:10.1371/journal.pone.0169588)

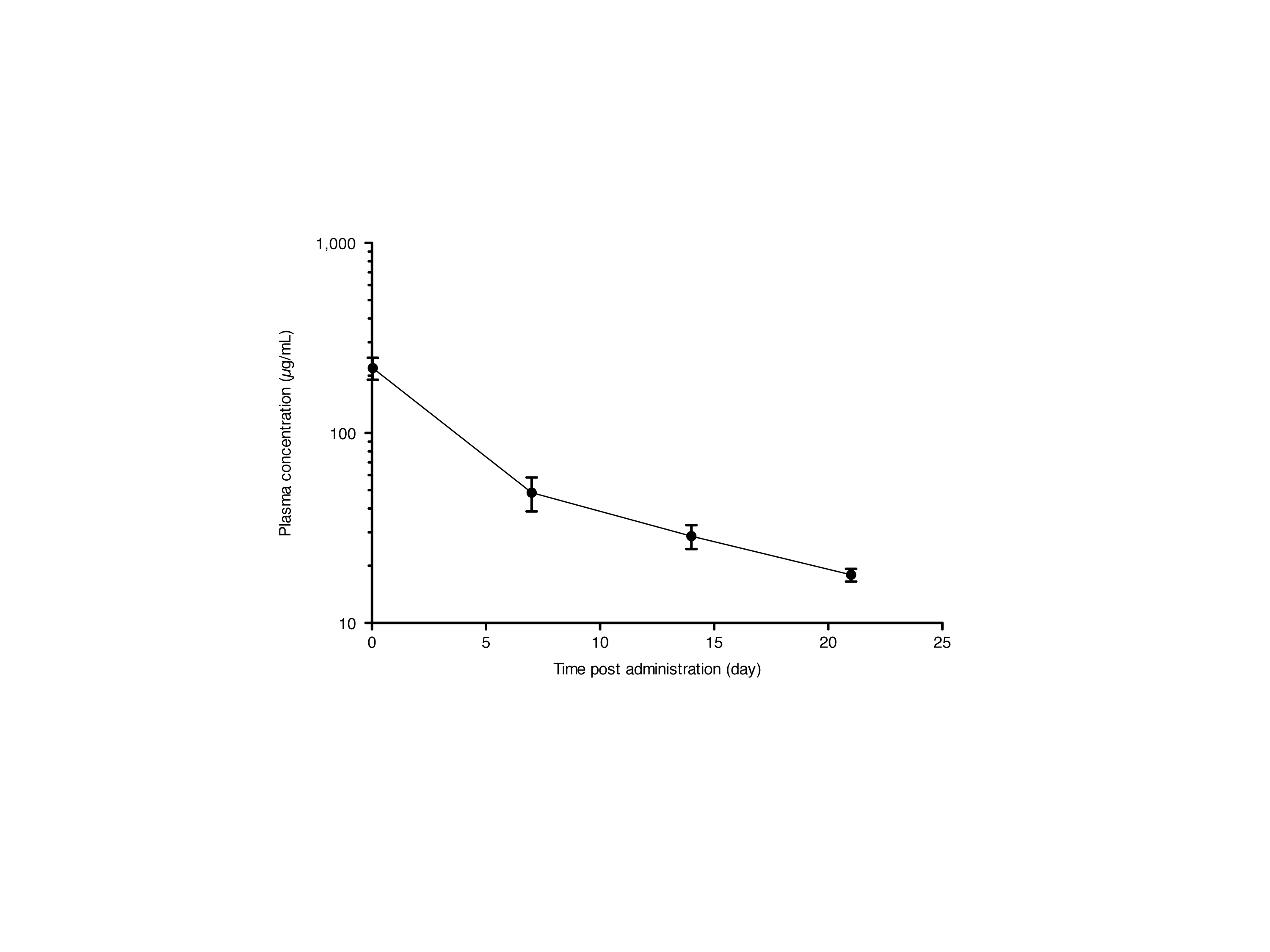

Supplement: S1 Fig — Rituximab (10 mg/kg) was administered to anesthetized rats at 10 mg/kg via the jugular vein. The concentration was determined by ELISA coated with anti-rituximab antibody (MB2A4). Each datum point represented the mean of six independent replicates with standard deviation. (TIF) [file pone.0169588.s001.tif]

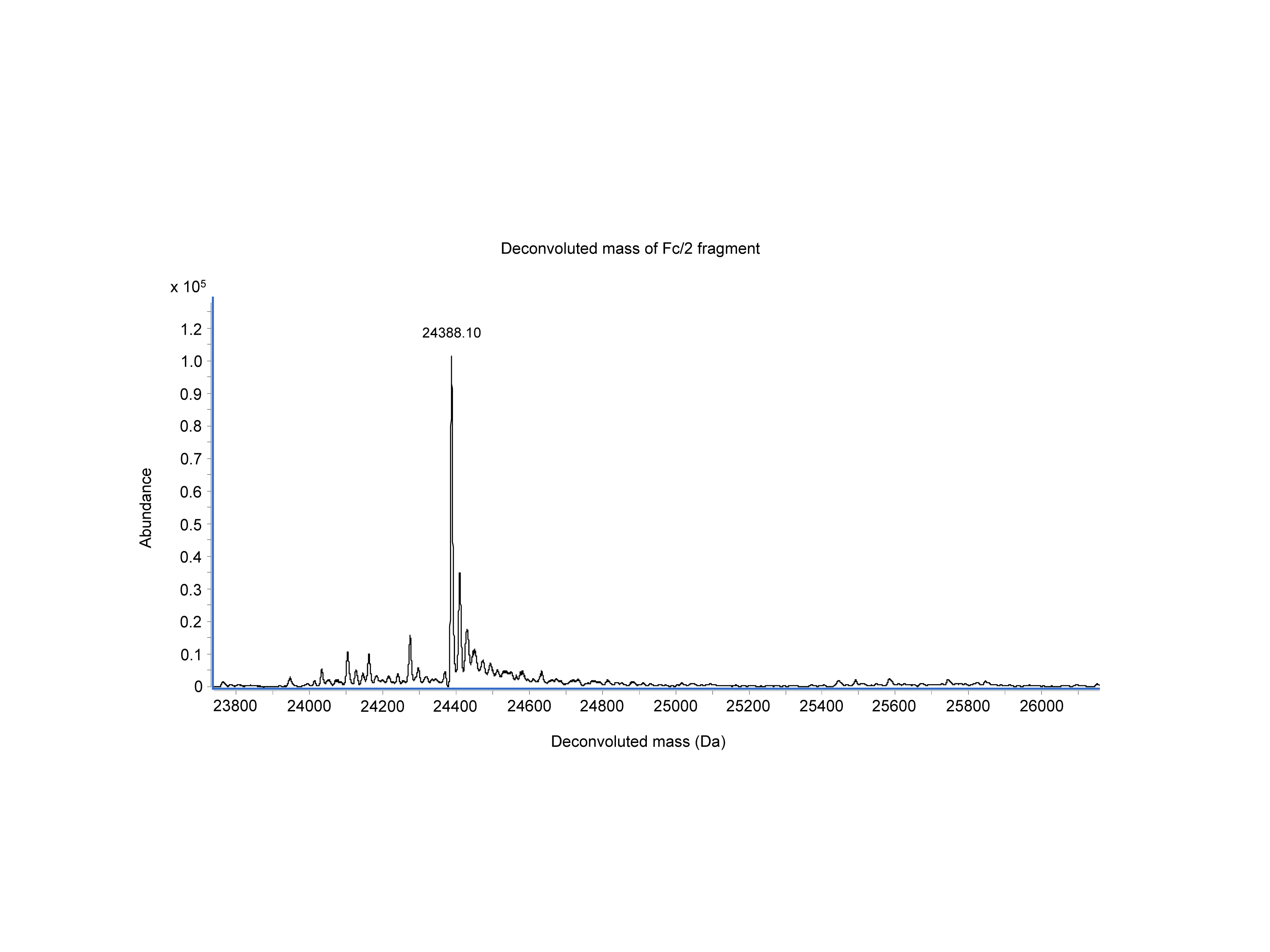

Supplement: S2 Fig — Rituximab in the formulation was deglycosylated for 1 h by EndoS. Papain digestion was conducted prior to LC/TOF-MS analysis. The most abundant ion in this spectrum was a deglycosylated Fc/2 fragment of rituximab. (TIF) [file pone.0169588.s002.tif]

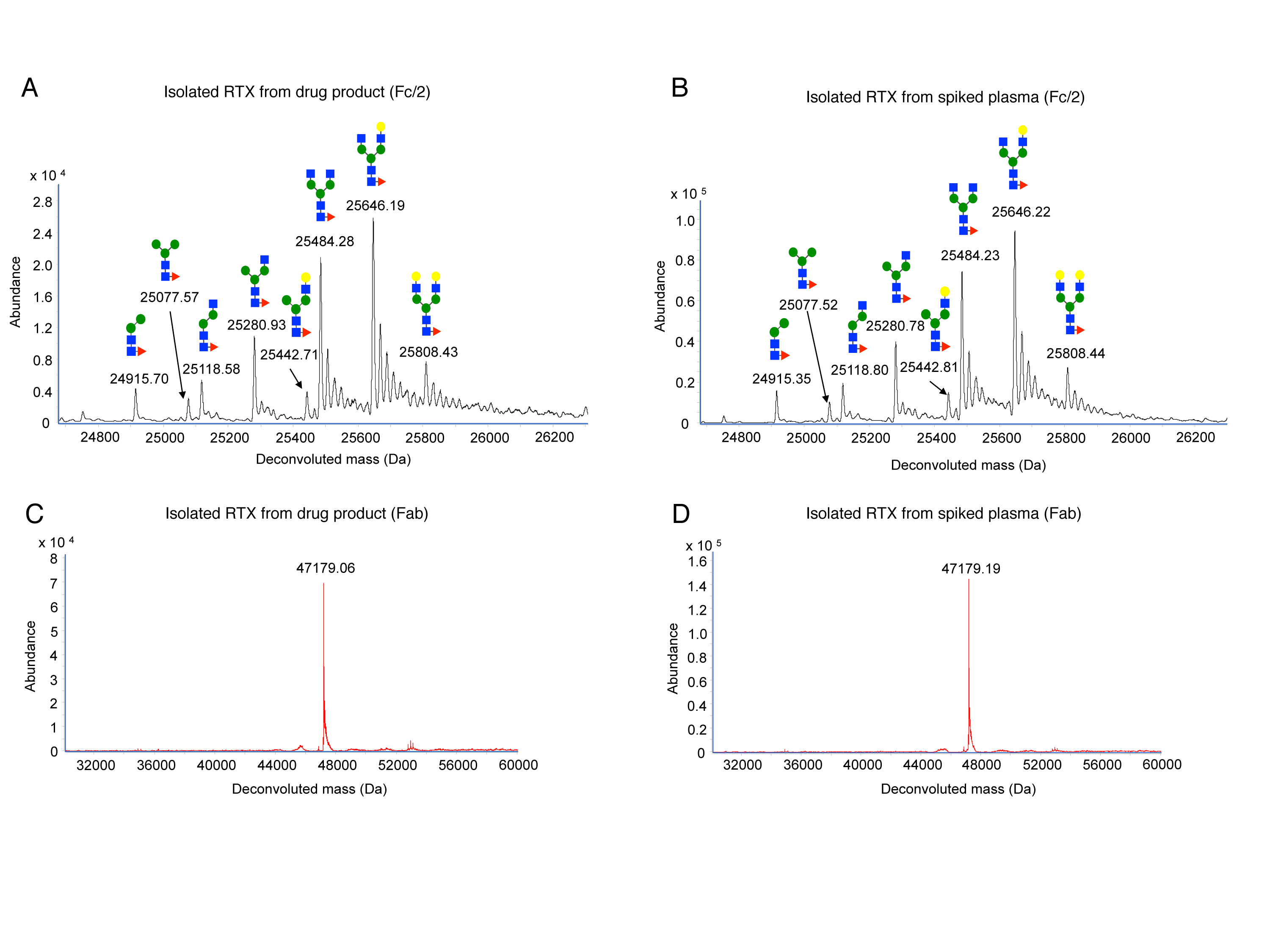

Supplement: S3 Fig — (A, B) Typical deconvoluted mass spectra of Fc/2 fragments of rituximab isolated from the commercial formulation and spiked plasma. The number and pattern diagram above each peak indicate observed molecular weights and predicted structures of attached carbohydrate chains, respectively. (C, D) Typical deconvoluted mass spectra of deglycosylated Fab fragments of rituximab isolated from the commercial formulation and spiked plasma. The most abundant ion in each spectrum was the Fab fragment of rituximab. (TIF) [file pone.0169588.s003.tif]

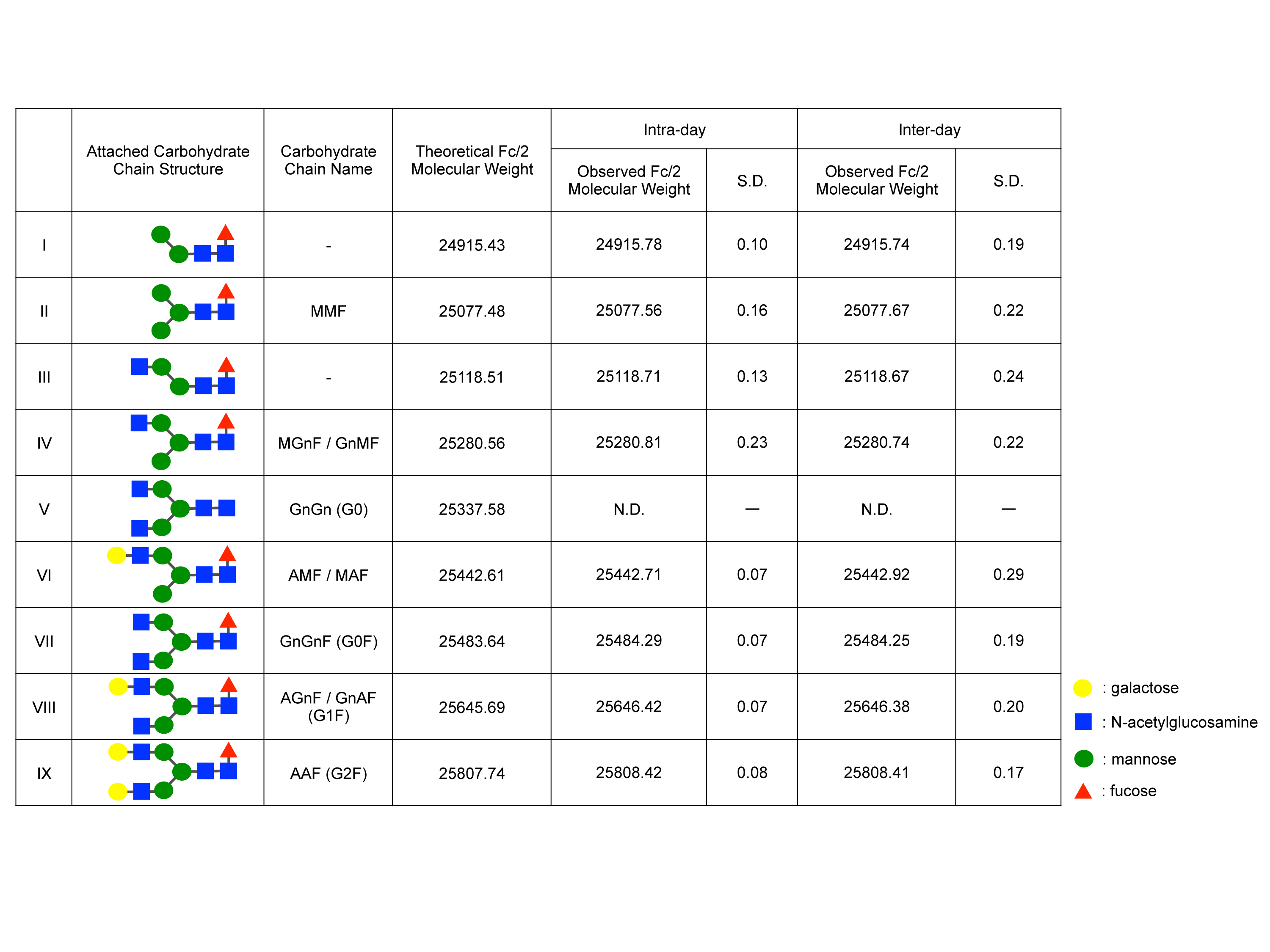

Supplement: S4 Fig — Observed Fc/2 molecular weights were the mean values of three independent experiments and the standard deviations of the experiments are given. Detected glycoforms in the rituximab formulation and the predictive attached carbohydrate chains were described in the same way as in Fig 3. (TIF) [file pone.0169588.s004.tif]
